# Supplementary material for: Dendrimer‐Like Supramolecular Assembly of Proteins with a Tunable Size and Valency Through Stepwise Iterative Growth
Source: Adv Sci (Weinh). 2021 Oct 31;8(24):2102991. doi: 10.1002/advs.202102991 (PMC8693032; doi:10.1002/advs.202102991)
Supplement: Supplementary file 1 — Supporting Information [file ADVS-8-2102991-s001.pdf]

ADVANCED SCIENCE
Open Access

Supporting Information

for *Adv. Sci.*, DOI: 10.1002/advs.202102991

Dendrimer-Like Supramolecular Assembly of Proteins with a Tunable Size and Valency Through Stepwise Iterative Growth

*Jin-Ho Bae, Hong-Sik Kim, Gijeong Kim, Ji-Joon Song, and Hak-Sung Kim**

Experimental Section

Protein expression and purification

All genes encoding the proteins used in the present work were cloned into a pET21a vector (Novagen) using NdeI and XhoI restriction sites. The genes for SpyCatcher and SnoopCatcher were synthesized as a gBlock Gene Fragment from Integrated DNA Technologies (IDT). SpyTag and SnoopTag were amplified using polymerase chain reaction. The protein monomers were linked to each other with a flexible linker (GSAGSAAGSGEF).[1] For the construction of an intercellular protein translocation module, the gene coding for an EGFR-targeting repebody, an off-target repebody, the translocation domain of *Pseudomonas aeruginosa* exotoxin, gelonin, and eGFP were amplified using PCR. Each protein domain was connected using a flexible GGGS linker, and a 6xHis-Tag was fused to the C-terminal of each construct. Amino acid sequences of proteins used for the construction and functionalization of protein assemblies are shown in **Table S1,** S2, **Supporting Information**.[2-4] For protein expression, the vectors harboring the gene were transformed into *E. coli* BL21 (DE3) and grown at 37 °C. When the optical density reached 0.6–0.8 at 600 nm, IPTG (0.5 mM) was added for induction. The induced cells were further grown for 18 h at 18 °C and harvested through centrifugation at 8000 rpm.

For protein purification, the harvested cells were resuspended in a lysis buffer (50 mM Tris, 300 mM NaCl, and 10 mM Imidazole at pH 7.8) and disrupted through sonication. Following centrifugation at 18,000 rpm for 1 h, the supernatant was loaded into a Ni-NTA Superflow (Qiagen). The loaded Ni-NTA column was washed with a wash buffer (50 mM Tris, 300 mM NaCl, and 20 mM Imidazole at pH 7.8). Finally, the proteins were eluted with an elution buffer (50 mM Tris, 300 mM NaCl, and 250 mM Imidazole at pH 7.8). All eluted proteins were further purified through size exclusion chromatography (Superdex 200, GE Healthcare). The monomers for protein assembly were purified using a sodium borate buffer (50 mM at pH 10.5), and constructs of gelonin and eGFP cargo were purified using a 20 mM Tris buffer (300 mM NaCl at pH 7.8).

Cell Culture

A431 (human epidermoid carcinoma, ATCC No. CRL-1555) and MCF7 (human adenocarcinoma, ATCC No. HTB-22) were cultivated in an RPMI medium (Capricorn) supplemented with 10% (v/v) fetal bovine serum (FBS, Hyclone). MDA-MB-468 (human

adenocarcinoma, ATCC No. HTB-132) and NIH3T3 (mouse embryonic fibroblast, ATCC No. CRL-1658) were cultivated in DMEM (Capricorn) supplemented with 10% (v/v) FBS. All cell cultures were carried out in a 5% CO_2 chamber at 37 °C.

Construction and purification of protein assembly

For the protein assembly, the core protein ($pG_{0,}$ zeroth-generation protein dendrimer) was incubated with an excess of 2–4 molar ratio equivalent of the total available termini using a building block protein in 50 mM sodium borate buffer (pH 9.0, 0.1% Tween-20). The resulting first-generation protein dendrimer (pG_1) was purified using SEC (Superdex 200, GE Healthcare) in a 50 mM sodium borate buffer (pH 10.5). The fractions for pG_1 were collected and concentrated using Amicon Ultra-15 Centrifugal Filters (Merck). For a further growth of the protein assembly, the same step was repeated using an alternate building block protein, yielding a protein dendrimer with a higher generation. For functionalization with protein cargos, each generation protein dendrimers were incubated using 3 molar ratio equivalent to the total available termini using a protein cargo which had been genetically fused to a conjugation module comprising a tandem of SpyTag and SnoopTag in a 20 mM Tris buffer (300 mM NaCl at pH 7.8) with 0.1% Tween-20. The functionalized protein dendrimers were purified using SEC (Superdex 200, GE Healthcare) in a 20 mM Tris buffer (300 mM NaCl at pH 7.8). For the analysis of the assembly and homogeneity of protein dendrimers, a Superdex Increase 200 10/300 (GE Healthcare) column was applied.

Confocal microscopy

For confocal imaging of the cells treated with protein dendrimers, the respective cells were attached to a microscopy 8-well chamber slide (SPL) at a density of 2×10^3 cells/well. After 3 days of growth, the serum-containing medium was treated with an appropriate amount of protein dendrimers at each generations for 6 h. To track the lysosome and acidic compartments, 200 nM of LysoTracker Red DND-99 (Thermo Scientific) was treated for 2 h. The cells were washed with DPBS containing 0.1% Tween-20 and fixed with 4% (v/v) paraformaldehyde for 20 min. Cell nuclei were counterstained with DAPI in the mounting medium (Vector). Confocal images were obtained using a Zeiss LSM 780 confocal microscope with a ×40 Apochromat objective with a 1.0 numerical aperture (Carl Zeiss).

In vitro cytotoxicity

Cells were seeded in a 96-well plate (SPL) at a density of 5×10^3 cells/well in a medium containing FBS. After growth overnight, the medium was changed to a fresh medium without serum, and an appropriate concentration of protein dendrimers carrying a protein cargo were added. After incubation for 12 h, the medium was replaced with a fresh medium without serum followed by further incubation for 3 days. Each well was supplemented with 10 μL of a CCK-8 reagent (Dojindo) and incubated for 2 h at 37 °C. Cell viability was determined using an Infinite M200 plate reader (Tecan) by measuring the OD_{450}.

Western blot assay

The cells were seeded in a 24 well plate (SPL) at a density of 2×10^5 cells/well and grown overnight. The cells were then treated with an appropriate concentration of protein dendrimers for 6 h, followed by three washes with cold DBPS. The cells were homogenized in a RIPA Lysis and Extraction Buffer (Thermo Scientific) with mild agitation for 30 min at 4 °C. The cell suspension was centrifuged for 20 min at 12,000 rpm, and the supernatant was collected. A total of 80 μg of protein was loaded and separated using a 12% SDS-PAGE gel. The proteins on the gel were transferred to a nitrocellulose membrane (Bio-Rad) at 100 V for 2 h in ice. The membrane was first blocked with PBS containing 0.1% Tween-20 and 3% (v/v) bovine serum albumin (BSA). Anti-GFP monoclonal (Santa Cruz) and anti-beta-actin monoclonal (Santa Cruz) primary antibodies were treated at 4 °C overnight, followed by incubation with HRP-conjugated anti-mouse IgG (Bio-Rad) secondary antibody at 37 °C for 1 h. The membrane was washed with PBST three times after each step and visualized using an enhanced chemi-luminescence solution (Millipore) with ChemiDoc XRS+ (Bio-Rad).

Dynamic light scattering (DLS)

Protein dendrimers were diluted in a PBS buffer (pH 7.5) and subjected to dynamic light scattering (DLS) to measure the hydrodynamic radii using a Zetasizer nano zs (Malvern). All measurements were taken at 25 °C.

Binding kinetics using surface plasmon resonance (SPR)

The association and dissociation kinetics of the functionalized protein dendrimers with an EGFR-targeting protein binder were determined through SPR using a BIACORE 3000 (Biacore AB). Human EGFR ectodomain with an hFc Tag (Sino Biological) was immobilized on a CM5 sensor chip through EDC/NHS conjugation chemistry. A mixture of 0.4 M 1-(3-dimethylaminopropyl)-3-ethylcarbodiimide hydrochloride (EDC) and 0.1M N-hydroxysulfosuccinimide (sulfo-NHS) was used to activate the chip surface, and 20 μg/mL of a human EGFR ectodomain in a sodium acetate buffer (10 mM at pH 4.5) was injected for immobilization onto the chip. Ethanolamine (1.0 M at pH 8.0) was used to block the chip. The functionalized protein dendrimers were injected at different concentrations. The sensorgrams were fit using a 1:1 Langmuir binding model using the software provided, and the association and disassociation rates were determined.

Multi-angle light scattering (MALS)

The absolute molecular masses of proteins were determined using multi-angle light scattering (MALS) (Dawn Heleos II, Enhanced Optical Signals) coupled to a Superdex 200 Increase 10/300 column (GE Healthcare). The absolute molecular masses were determined using the ASTRA program (Wyatt Technologies).

Transmission electron microscopy (TEM)

For negative stain TEM imaging, protein dendrimer samples (0.05mg/ml) were applied to glow-discharged carbon grids and incubated for 1 min. The grids were then washed twice with water and incubated with 1.5% (w/v) uranyl acetate for 1 min. The grids were blotted using filter paper and dried under ambient conditions. The prepared grids were analyzed using a Tecnai F20 200-kV field-emission transmission electron microscope (FEI) with a CCD camera (Gatan).

Statistical Analyses

All experiments were conducted in triplicate unless otherwise specified. All experimental results were presented as mean ± standard deviation (SD) (n = 3).

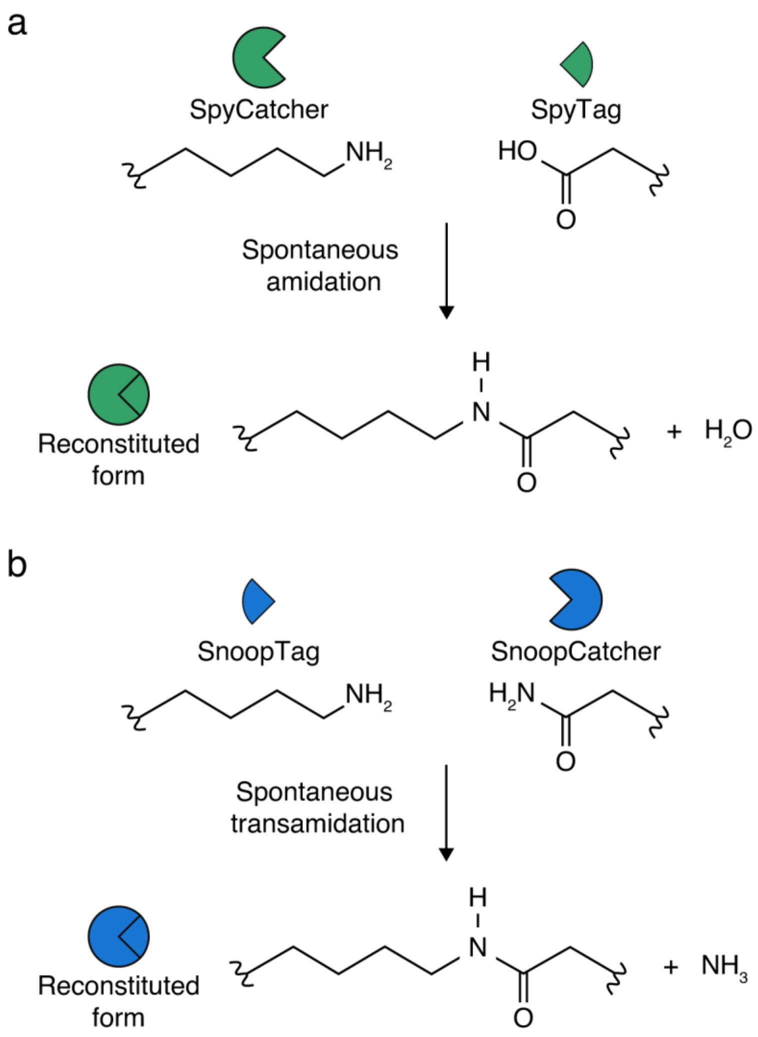

Scheme S1. Schematics for the isopeptide bond formation between a Catcher and a Tag. (a) Chemical reaction between a SpyCatcher and a SpyTag. Upon mixing, the Lys and Asp of the two domains spontaneously form an isopeptide bond releasing water. **(b)** Chemical reaction between a SnoopCatcher and a SnoopTag. Upon mixing, the Lys and Asn of the two domains spontaneously form an isopeptide bond releasing ammonia.

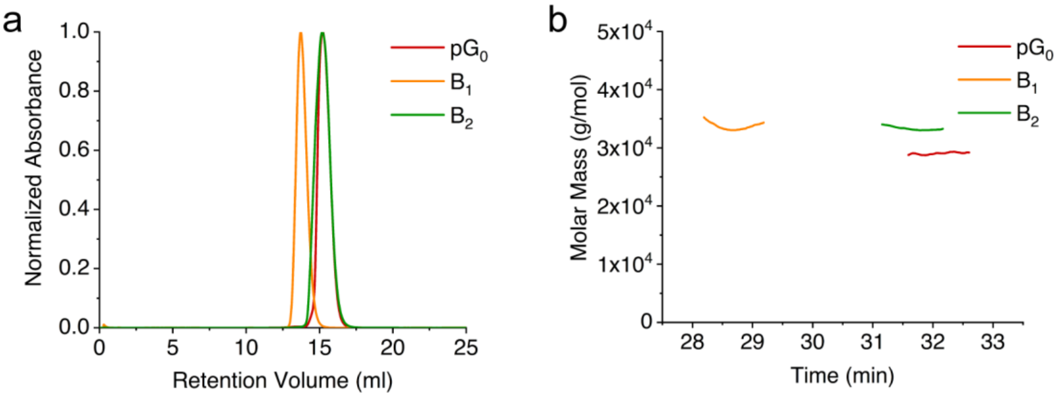

c

Monomer	Expected M_w (kDa)	Observed M_w (kDa)
pG_0	27.3	29.1
B_1	29.8	34.3
B_2	32.6	34.5

Figure S1. Purification and biophysical characterization of the core protein and two building blocks for the construction of protein dendrimers. (a) SEC of the core protein (pG_0) and two building blocks (B_1 and B_2). The peaks represent the normalized absorbance of each protein at 280 nm. **(b)** Absolute molecular masses of the core protein and two building blocks by MALS. Eluted protein fractions from SEC were analyzed. **(c)** Summary of the absolute molecular masses of the core and two building blocks determined by MALS in (b).

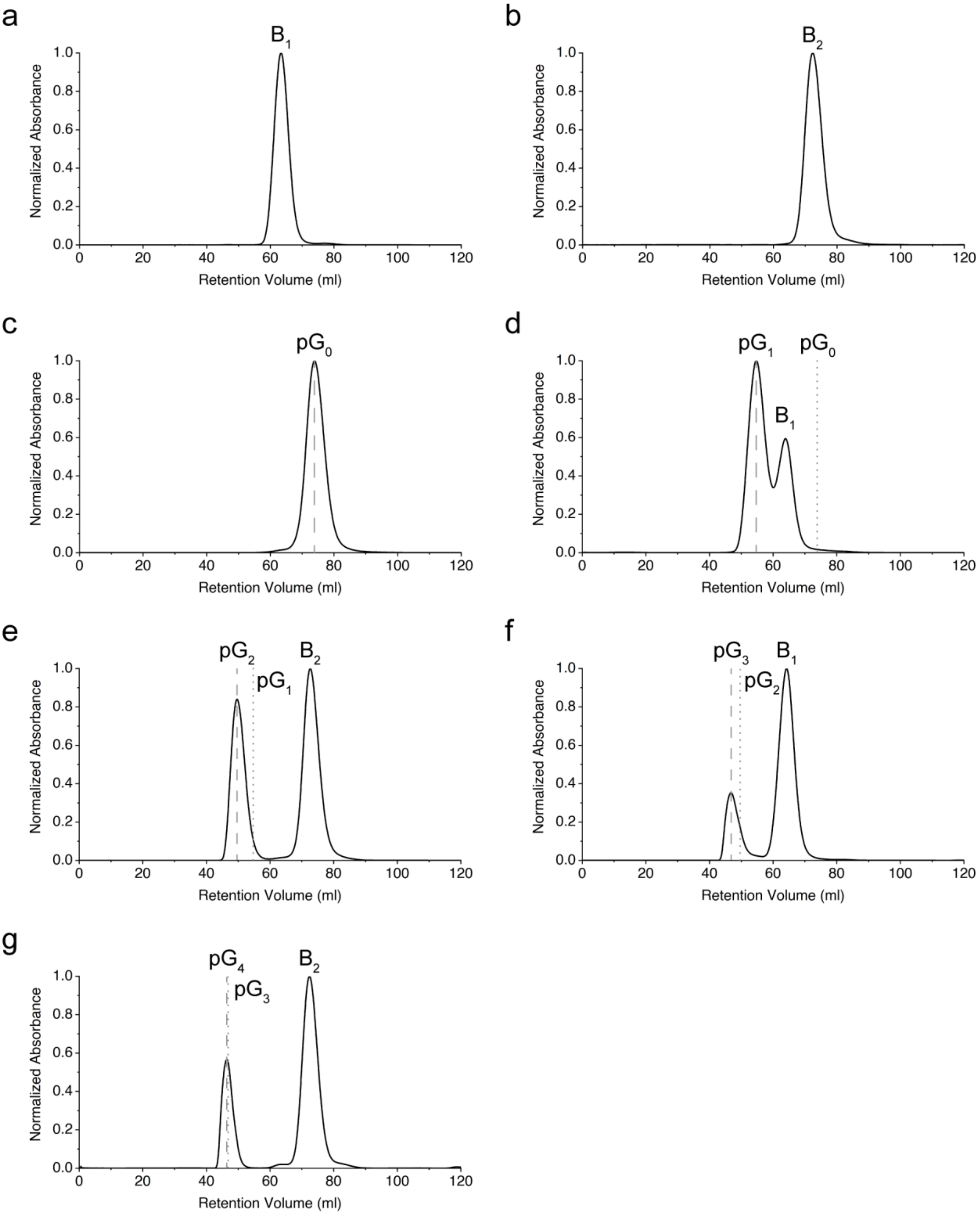

Figure S2. Construction of different protein dendrimer generations through a stepwise iterative growth. Different generations of protein dendrimers were purified by SEC using a S200 column in sodium borate buffer (pH 10.5). The purified protein dendrimers were used for the construction of the next generation protein dendrimers. Elution peaks were monitored at 280 nm. **(a)** Building block protein B_1. **(b)** Building block protein B_2. **(c)** Zeroth-generation protein dendrimer pG_0 (Core protein). **(d)** Construction and analysis of the first-generation protein dendrimer pG_1. The core protein pG_0 was incubated with an excess of a building

block protein B_1 overnight, followed by purification through SEC. The dotted line represents the peak maximum of the previous generation protein dendrimer (in this case pG_0), and the dashed line indicates the peak maximum of a next-generation protein dendrimer (in this case pG_1). All of pG_0 were efficiently grown to pG_1 by addition of B_2, leaving a negligible trace of the pG_0 peak. Eluted fractions of pG_1 were collected for further growth. (**e**) Construction and analysis of the second-generation protein dendrimer $pG_{2.}$ The growth procedure and analysis conditions were the same as in (d). **(f)** Construction and analysis of the third-generation protein dendrimer $pG_{3.}$ **(g)** Construction and analysis of the fourth-generation protein dendrimer pG_4. In the case of pG_3 and $pG_{4,}$ both proteins were eluted near the void fractions due to their large molecular masses, resulting in the overlap of the peak maximum.

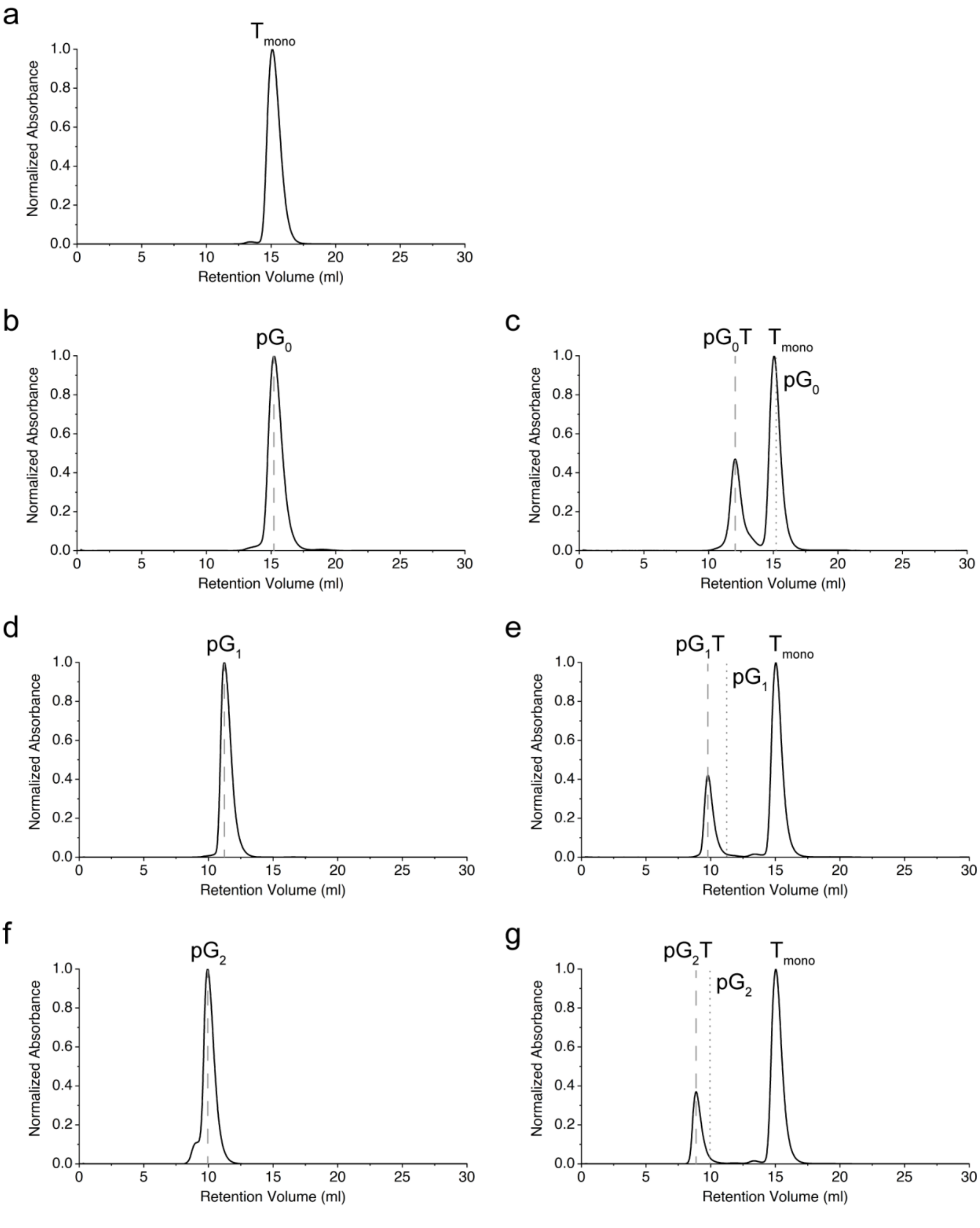

Figure S3. Functionalization of protein dendrimers with a targeting moiety and analysis of the functionalized protein dendrimers through SEC. The peaks in SEC show the normalized absorbance of the functionalized protein dendrimers at different generations at 280 nm using a S200 column in Tris buffer (pH 7.5). Void volume is 8.48 ml. **(a)** Purification of an EGFR-specific repebody fused to a conjugation module, T_{mono}. The conjugation module comprises of a tandem of SpyTag and SnoopTag linked to each other using a GS linker. **(b)** Zeroth-generation protein dendrimer pG_0. **(c)** Formation of pG_0T by incubating pG_0 with an

excess of T_{mono} overnight. The dotted line represents the peak maximum of the starting protein dendrimer (in this case pG_0), and the dashed line indicates the peak maximum of the newly functionalized protein dendrimer (in this case pG_0T). (**d**) Purified $pG_{1.}$ **(e)** Formation of pG_1T by incubating pG_1 with an excess of T_{mono} overnight. **(f)** Purified pG_2. **(g)** Formation of pG_2T by incubating pG_2 with an excess of T_{mono} overnight.

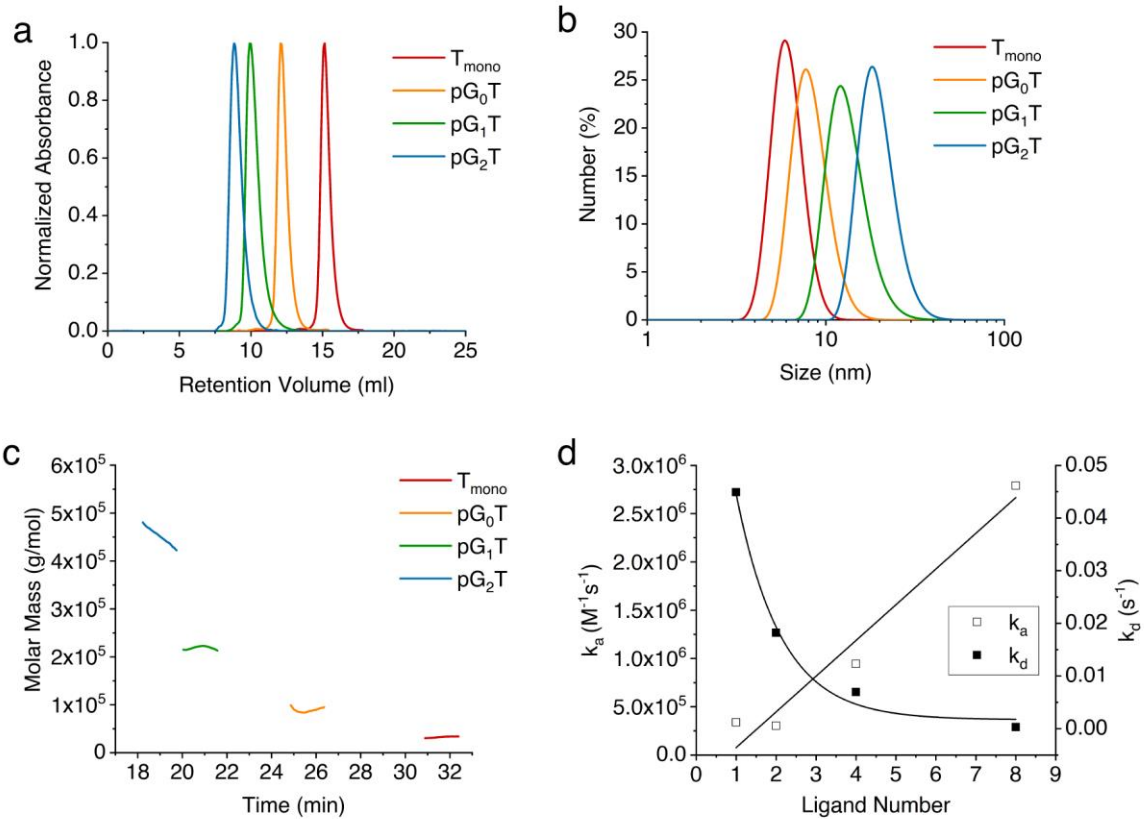

Figure S4. Biophysical characterization of the protein dendrimers functionalized with a target-specific protein binder. (a) SEC of T_{mono} and different generation protein dendrimers functionalized with an EGFR-specific repebody. The peaks represent the normalized absorbance of each generation at 280 nm. **(b)** DLS of the protein dendrimers functionalized with the repebody. **(c)** Absolute molecular masses of the protein dendrimers functionalized with the repebody by MALS. The protein dendrimers eluted from SEC were analyzed. **(d)** Association and dissociation rates of the functionalized protein dendrimers based on the sensorgrams represented in **Figure 2e** as a function of the number of the EGFR-specific repebody molecules.

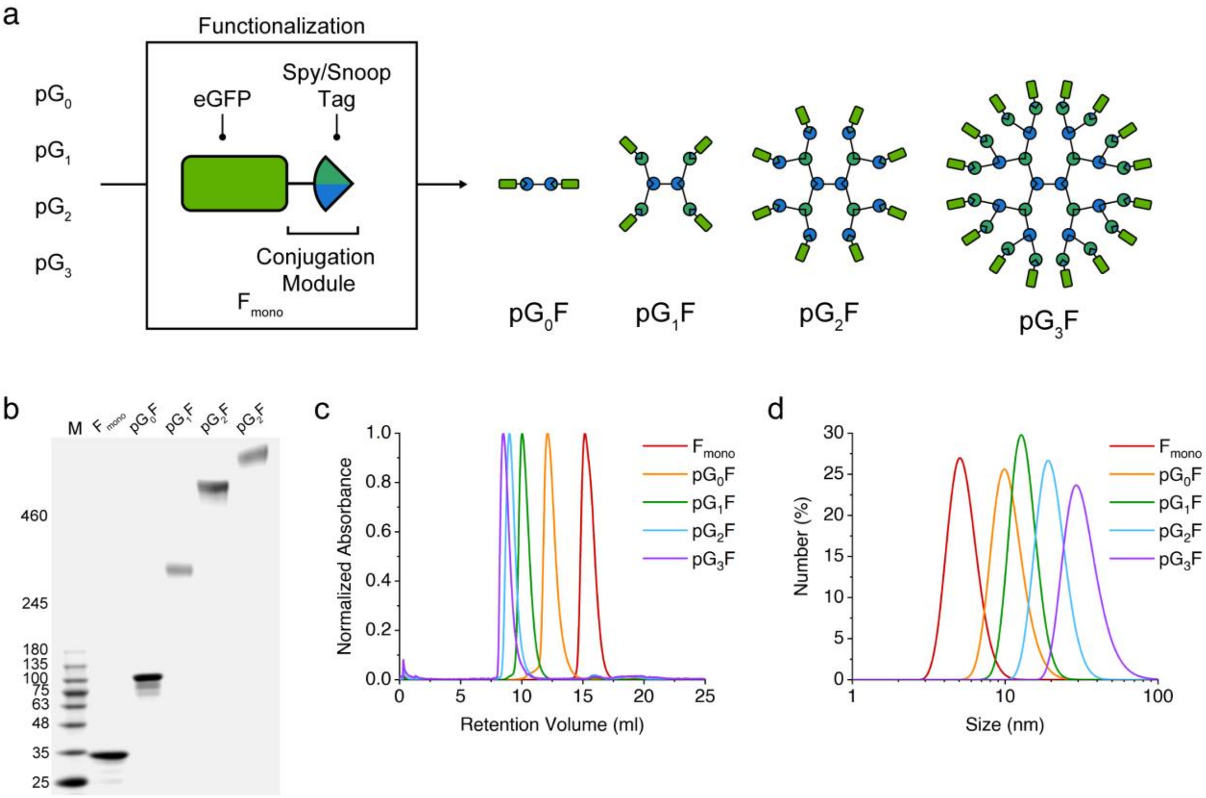

e

Construct	No. of Cargo	Expected M_w (kDa)	R_h (nm)	Average Diameter (nm)
F_{mono}	1	32.6	5.36	-
pG_0F	2	92.5	10.67	9.43
pG_1F	4	222.9	13.29	13.87
pG_2F	8	472.8	20.26	21.63
pG_3F	16	994.2	32.83	28.10

Figure S5. Functionalization of protein dendrimers with eGFP and biophysical characterization of the functionalized protein dendrimers. (a) Graphical representation of the functionalization of different generation protein dendrimers with eGFP which had been genetically fused to the N-terminal of a conjugation module to produce F_{mono}. The conjugation module comprises of a tandem of SpyTag and SnoopTag linked to each other using a GS linker. Using either SpyCatcher or SnoopCatcher at the periphery depending on the generation, the protein dendrimers were functionalized with eGFP, yielding pG_0F, pG_1F, and pG_2F and pG_3F. **(b)** SDS-PAGE (3%–12% gradient) of the protein dendrimers functionalized with eGFP. **(c)** SEC of the functionalized protein dendrimers with eGFP. The elution peaks represent the normalized absorbance at 280 nm. **(d)** DLS analysis of the protein dendrimers functionalized with F_{mono}. **(e)** Summary of the expected molecular masses, DLS measurements and the average diameter as shown in **Figure 2h** of different protein dendrimer generations functionalized with $F_{mono.}$

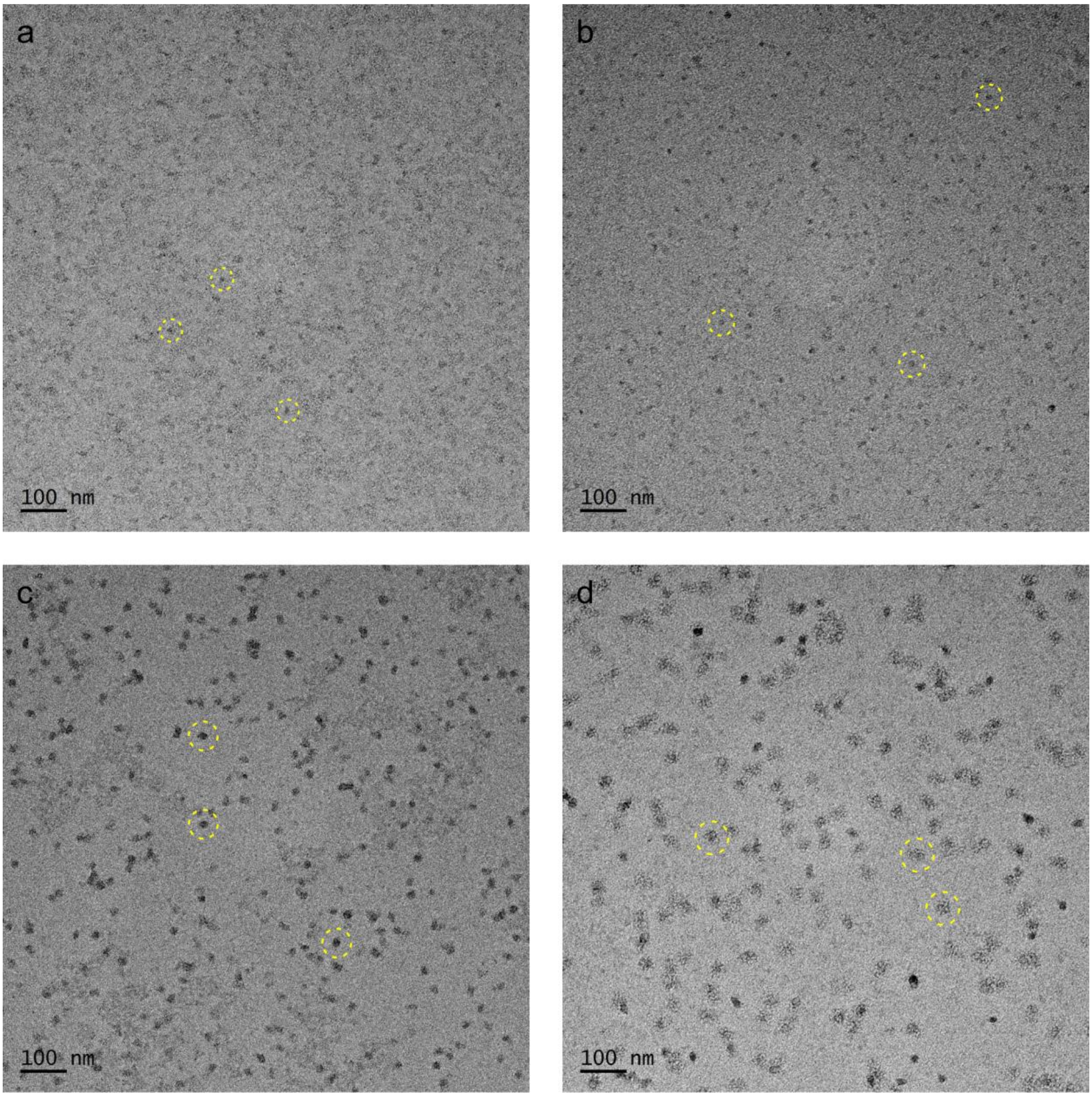

Figure S6. TEM image of the protein dendrimers functionalized with eGFP. Different generations of protein dendrimers were functionalized with eGFP and subjected to TEM analysis**. (a)** pG_0F. **(b)** pG_1F. **(c)** pG_2F. **(d)** pG_3F. The images in yellow dashed circles indicate representative protein dendrimers.

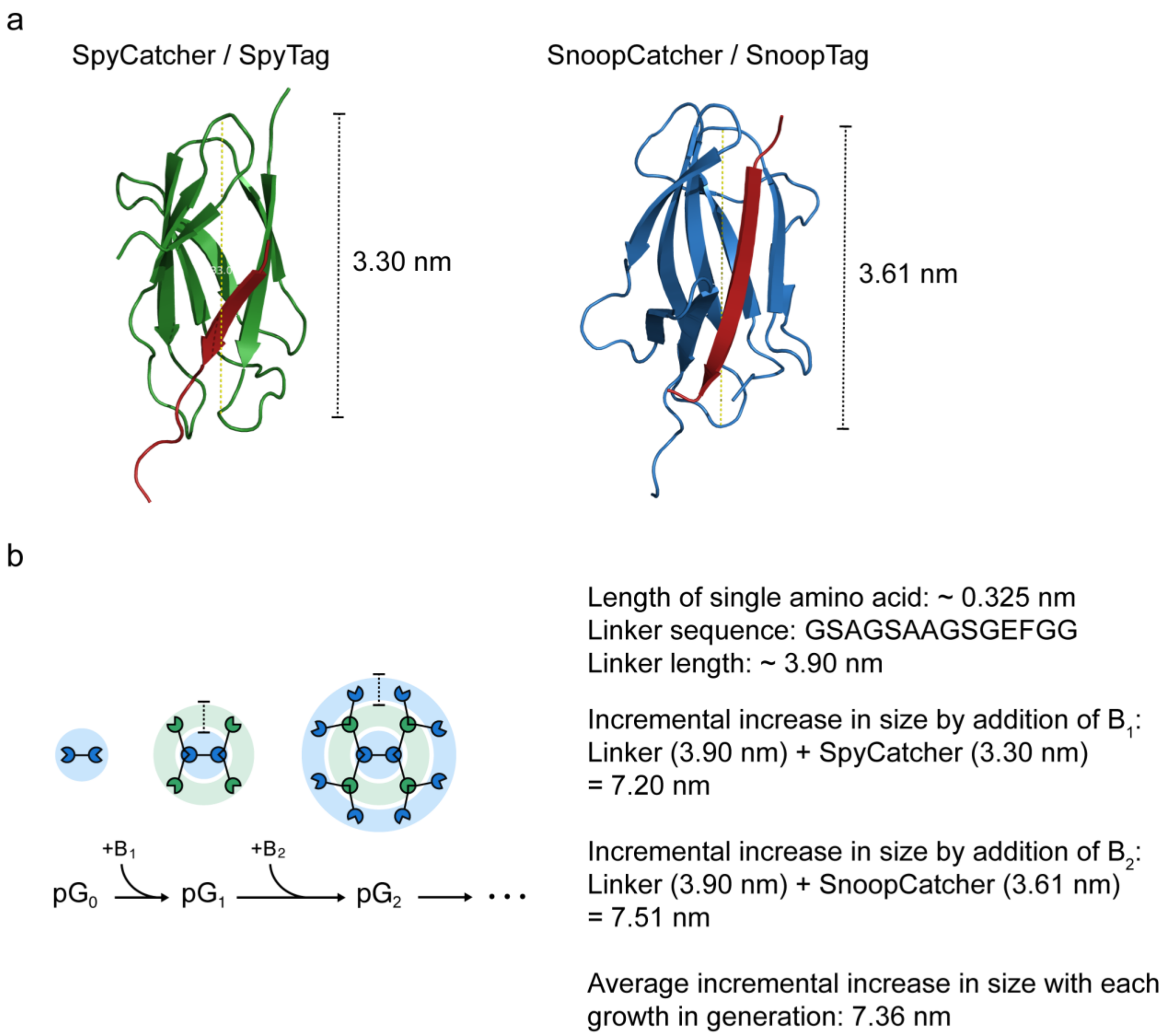

Figure S7. Approximate incremental increase in the size of protein dendrimers with the increasing generation. (a) Structure and size of a SpyCatcher/SpyTag (PDB ID: 4MLI) and a SnoopCatcher/SnoopTag (PDB ID: 2WW8) represented by PyMOL. SpyCatcher and the reconstituted SpyTag are indicated by green and red, respectively. SnoopCatcher and the reconstituted SnoopTag are represented by blue and red, respectively. **(b)** According to **Scheme 1**, the SpyTag/SnoopTag at the middle of a building block is linked to the previous generation protein dendrimer. Approximate length of a linker between each building block protein, given that each amino acid is ≈0.325 nm and the linker is fully extended, is 3.90 nm.[5] Thus, the increase in the average size in the next generation protein dendrimer corresponds to the sum of the length of a linker and a single SpyCatcher/SnoopCatcher protein. The maximum growth in the size of protein dendrimer is an average of ≈7.36 nm, assuming that the growth is one-dimensional. The theoretical increase in the size of protein dendrimer with each generation matches the incremental increase of 6.2 nm, which was estimated using TEM in **Figure 2h**, considering that the protein in solution are three-dimensional and not completely extended.

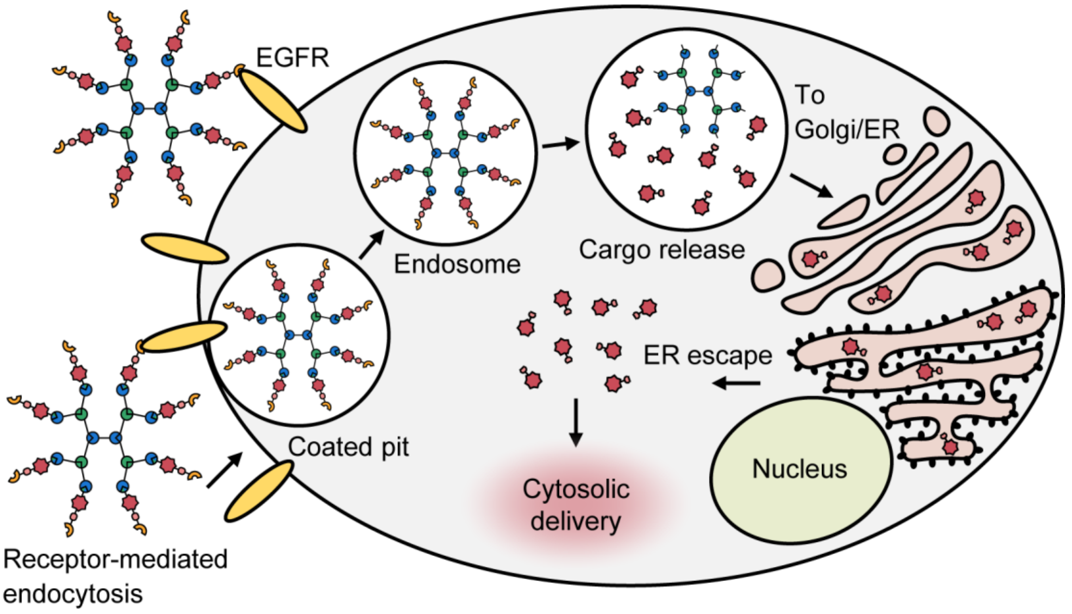

Figure S8. Scheme of intracellular protein delivery using protein dendrimers. A protein cargo is delivered to the cytosol by the protein dendrimers functionalized with a targeting moiety and a protein cargo. An EGFR-specific repebody on the protein dendrimer binds to the cell surface EGFR, and the functionalized protein dendrimer undergoes endocytosis. In the endosome, the cargo is released through the cleavage of TDP by furin and cathepsin. The cargo is then translocated to the ER by the KDEL receptor, followed by the release to the cytosol.

a

Construct	No. of Cargo	Expected M_w (kDa)	R_h (nm)
D_{mono} (+)	1	74.3	6.9
pG_0D (+)	2	176.0	12.3
pG_1D (+)	4	389.9	19.5
pG_2D (+)	8	806.6	26.9
D_{mono} (-)	1	76.8	6.9
pG_0D (-)	2	180.9	12.6
pG_1D (-)	4	399.7	19.4
pG_2D (-)	8	826.4	27.1

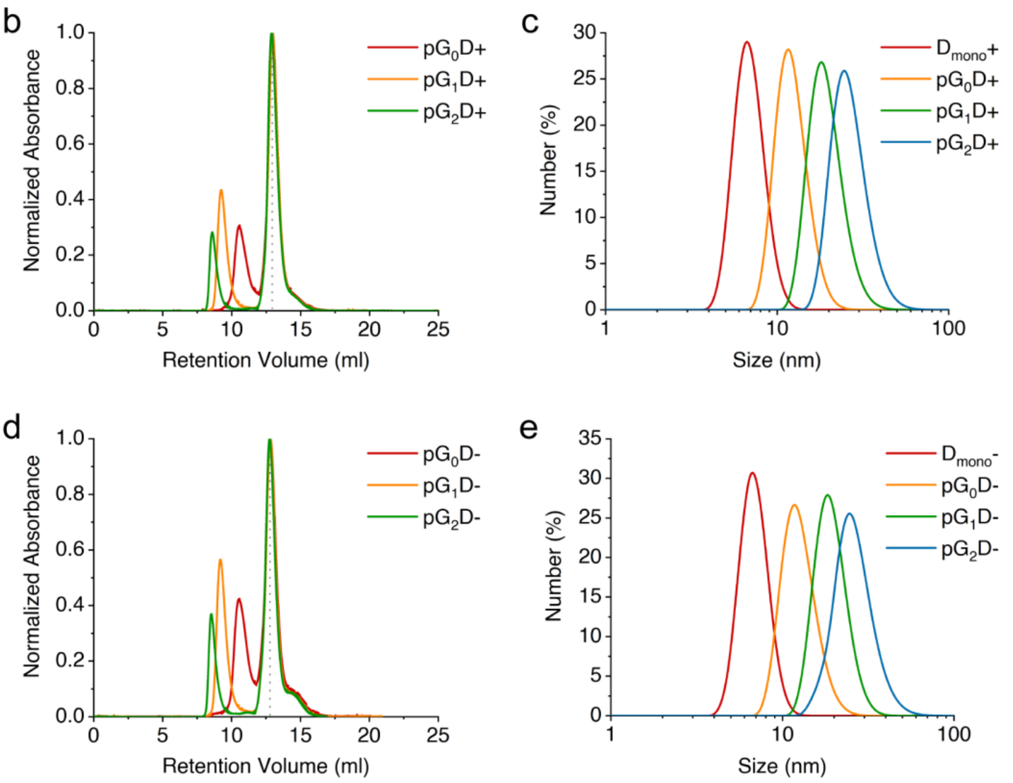

Figure S9. Biophysical characterization of the protein dendrimers functionalized with D_{mono}. (a) Summary of the expected molecular masses and the hydrodynamic radii of different protein dendrimer generations functionalized with D_{mono}. (+) and (-) indicate the translocation module carrying an EGFR-specific repebody and an off-target repebody, respectively. (**b**) SEC of the functionalized protein dendrimers with the translocation module carrying an EGFR-specific repebody. The peaks represent the normalized absorbance at 280 nm. The dotted line represent the excess D_{mono} (+) proteins used for functionalization. **(c)** DLS analysis of the protein dendrimers functionalized with the translocation module carrying an EGFR-specific repebody. (**d**) SEC of the protein dendrimers functionalized with the translocation module carrying an off-target repebody. The dotted line represent the excess D_{mono} (-) proteins used for functionalization. **(e)** DLS analysis of the protein dendrimers functionalized with the translocation module carrying an off-target repebody.

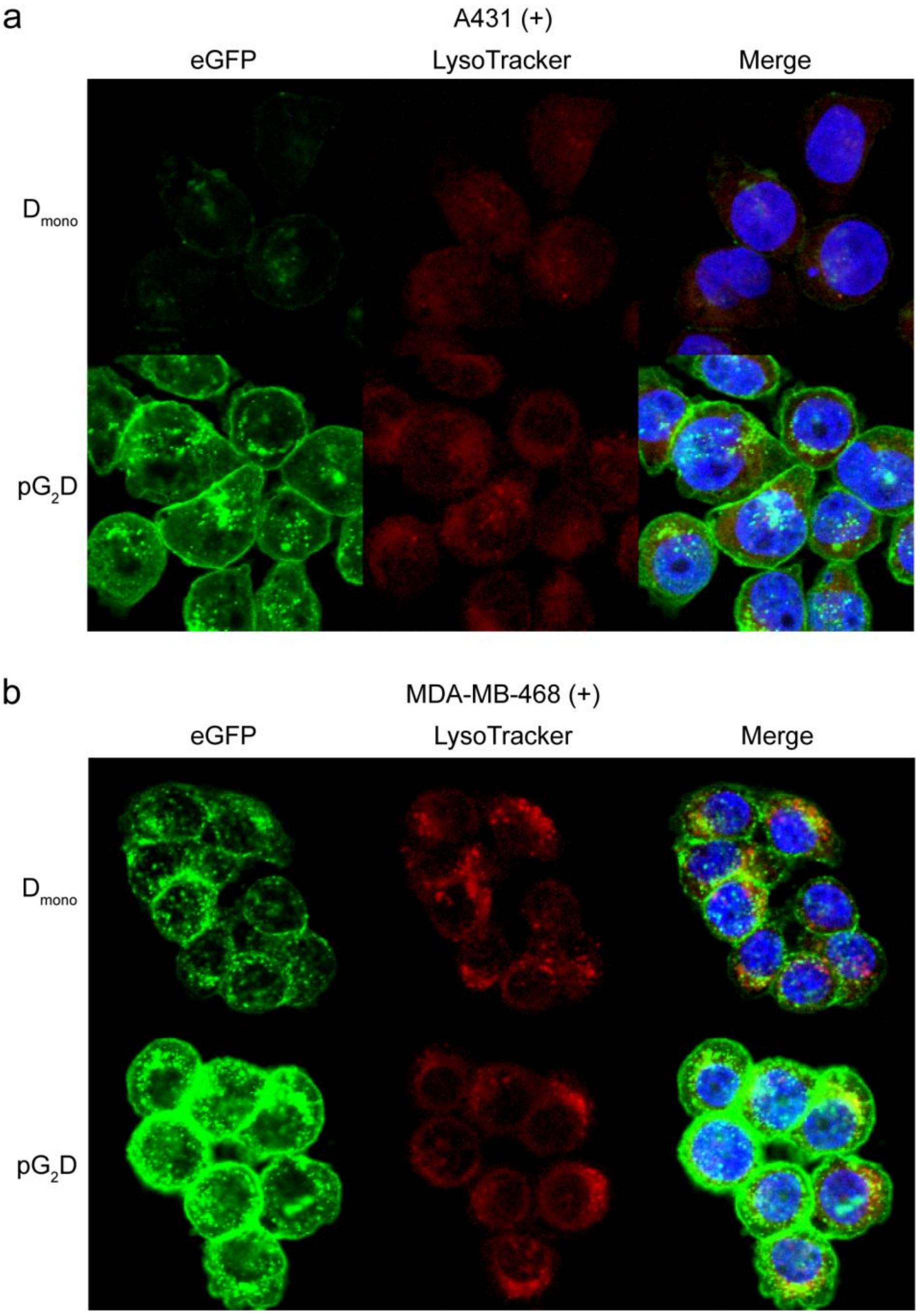

Figure S10. Enlarged confocal imaging of high EGFR-expression cell lines treated with $\mathbf{D_{mono}}$ and $\mathbf{pG_2D}$. (a) Confocal image of high EGFR-expressing A431 after treatment with the protein dendrimers functionalized with D_{mono} (+). Intracellular delivery of eGFP was traced with lysotrackers. **(b)** Confocal image of high EGFR-expressing MDA-MB-468 after treatment with the same protein dendrimers as in (a). All cells were treated for 6 h.

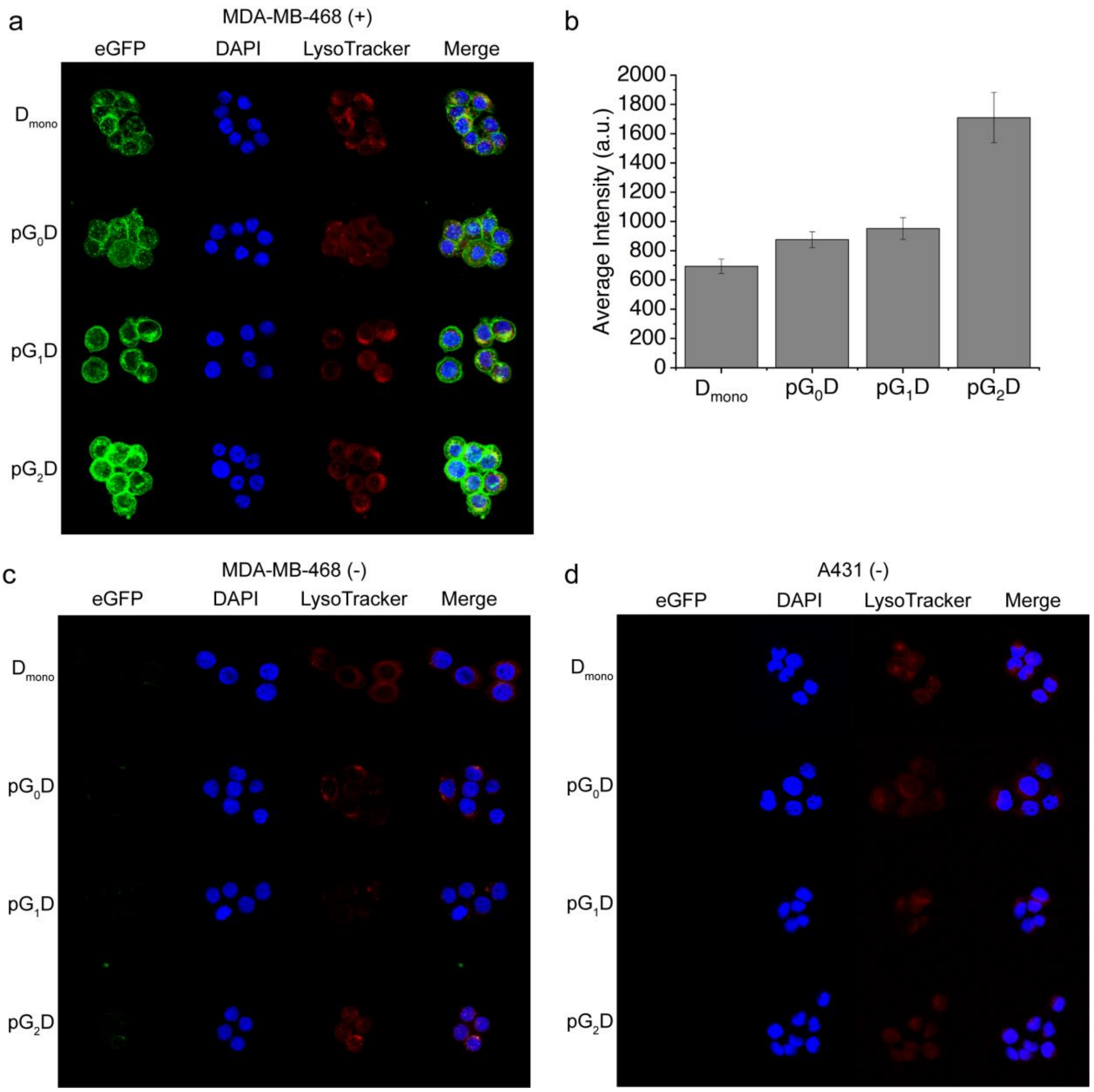

Figure S11. Confocal images of high EGFR-expressing cell lines treated with the protein dendrimers functionalized with D_{mono}. (a) Confocal images of high EGFR-expressing MDA-MB-468 cells after treatment with the protein dendrimers functionalized with D_{mono} (+). **(b)** Average cell fluorescence intensity of eGFP from MDA-MB-468 cells treated with the same protein dendrimers as in (a) (n = 5). **(c)** Confocal images of MDA-MB-468 cells after treatment with the protein dendrimers functionalized with D_{mono} (-). (**d**) Confocal images of A431 cells after treatment with the same protein dendrimers as in (c). All cells were treated for 6 h.

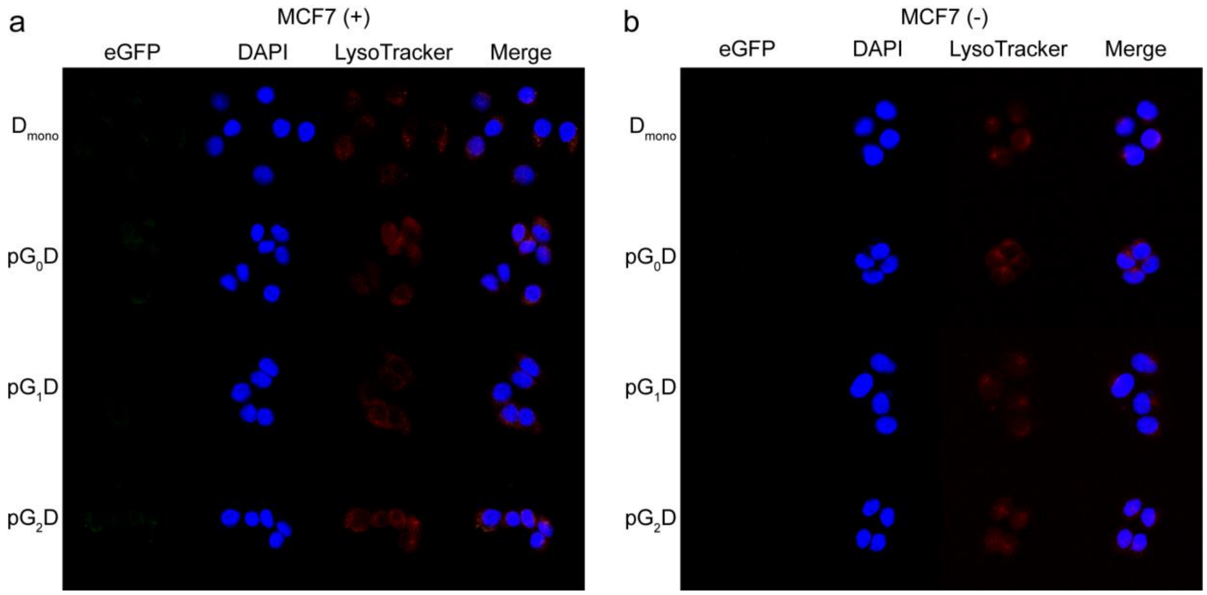

Figure S12. Confocal images of low EGFR-expressing MCF7 cell line treated with the protein dendrimers functionalized with D_{mono}. (a) Confocal images of MCF7 cells after treatment with the protein dendrimers functionalized with D_{mono} (+). **(b)** Confocal images of MCF7 cells after treatment with the protein dendrimers functionalized with D_{mono} (-). All cells were treated for 6 h.

a

Construct	No. of Cargo	Expected M_w (kDa)	R_h (nm)
G_{mono} (+)	1	75.7	6.9
pG_0G (+)	2	178.6	13.7
pG_1G (+)	4	395.1	21.6
pG_2G (+)	8	817.2	28.3
G_{mono} (-)	1	78.1	6.5
pG_0G (-)	2	183.6	12.8
pG_1G (-)	4	405.0	18.3
pG_2G (-)	8	836.9	25.2

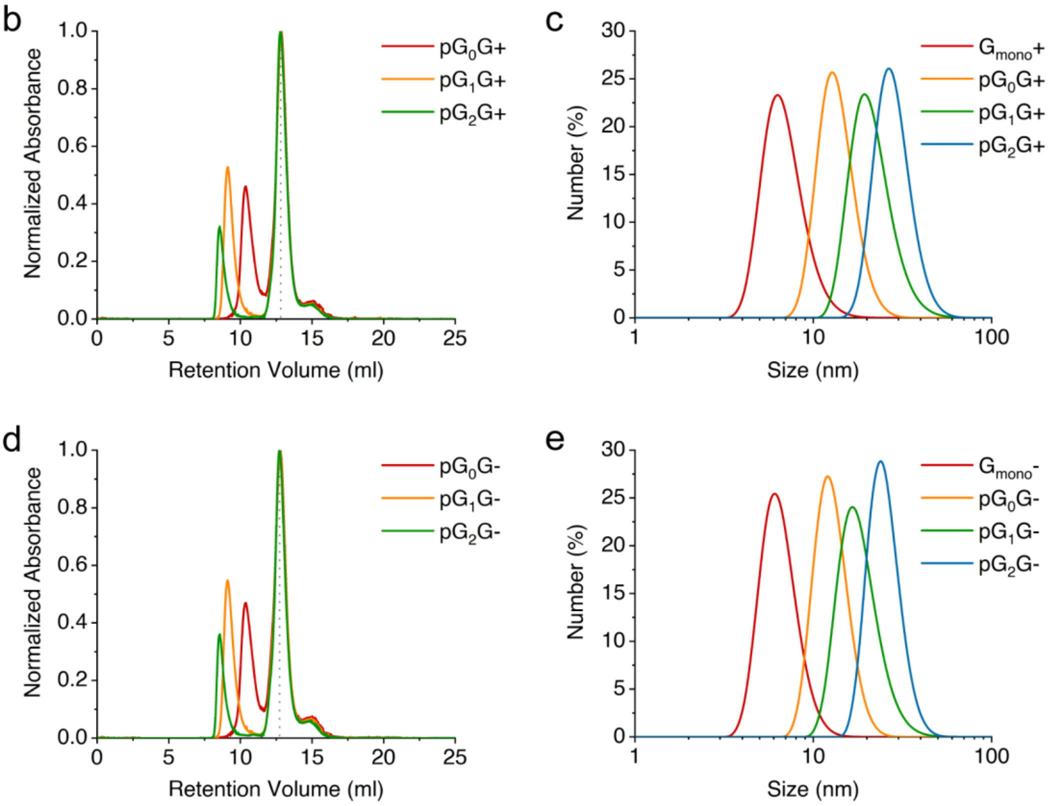

Figure S13. Biophysical characterization of the protein dendrimers functionalized with G_{mono}. (a) Summary of the expected molecular masses and the hydrodynamic radii of the protein dendrimers functionalized with gelonin. (+) and (-) indicate the functionalized protein dendrimers containing an EGFR-specific repebody and an off-target repebody, respectively. **(b)** SEC of the functionalized protein dendrimers containing an EGFR-specific repebody. The peaks represent the normalized absorbance at 280 nm. The dotted line represent the excess G_{mono} (+) proteins used for functionalization. **(c)** DLS analysis of the functionalized protein dendrimers containing an EGFR-specific repebody. **(d)** SEC of the functionalized protein dendrimers containing an off-target repebody. The dotted line represent the excess G_{mono} (-) proteins used for functionalization. **(e)** DLS analysis of the functionalized protein dendrimers

containing an off-target repebody.

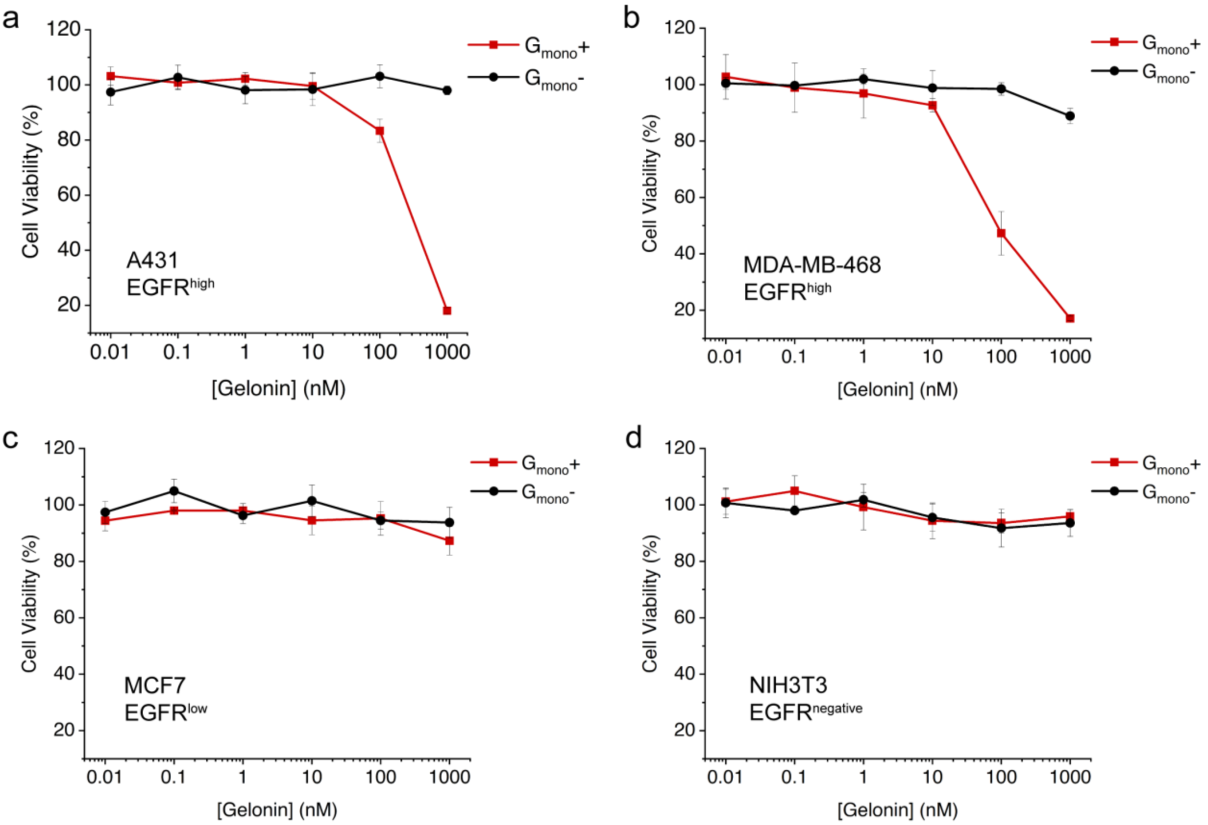

Figure S14. Viability of cells treated with G_{mono}. Cells were treated with G_{mono} containing an EGFR-specific repebody and an off-target repebody, respectively, for 12 hours, and cell viabilities were measured with respect to the G_{mono} concentration. (+) indicates G_{mono} carrying an EGFR-specific repebody, while (-) represents G_{mono} carrying an off-target repebody. **(a)** A431, **(b)** MDA-MB-468, **(c)** MCF7, **(d)** NIH3T3.

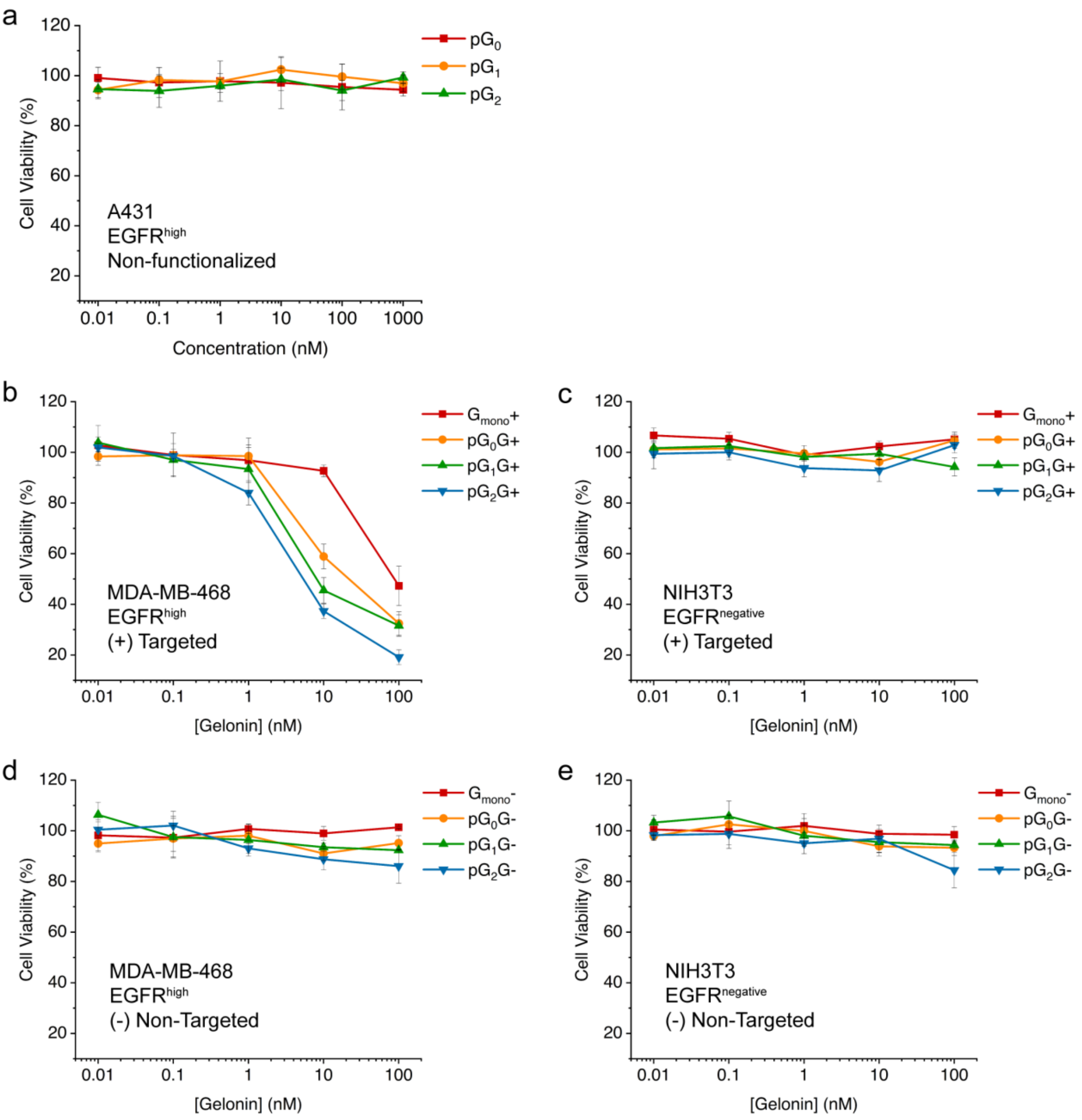

Figure S15. Viability of cells treated with different generation protein dendrimers functionalized with G_{mono}. (a) Viability of A431 cells treated with different generation protein dendrimers, pG_0 to pG_2. **(b)** Viability of MDA-MB-468 cells after treatment with the protein dendrimers functionalized with G_{mono} (+). **(c)** Viability of NIH3T3 cells after treatment with the functionalized protein dendrimers with G_{mono} (+). **(d)** Viability of MDA-MB-468 cells after treatment with the functionalized protein dendrimers with G_{mono} (-). **(e)** Viability of NIH3T3 cells after treatment with the functionalized protein dendrimers with G_{mono} (-). All cells were treated for 12 h.

Table S1. Amino acid sequences of the core protein (pG_0) and two building blocks (B_1 and B_2). Blue and green sequences indicate SnoopCatcher and SpyCatcher, respectively. Blue and green underlined sequences indicate SnoopTag and SpyTag, respectively.

**pG_0**<br>MKPLRGAVFSLQKQHPDYPDIYGAIDQNGTYQNVRTGEDGKLTFKNLSDGKYRLFE<br>NSEPAGYKPVQNKPIVAFQIVNGEVRDVTSIVPQDIPATYEFTNGKHYITNEPIPPKGSA<br>GSAAGSGEFGGKPLRGAVFSLQKQHPDYPDIYGAIDQNGTYQNVRTGEDGKLTFKNL<br>SDGKYRLFENSEPAGYKPVQNKPIVAFQIVNGEVRDVTSIVPQDIPATYEFTNGKHYIT<br>NEPIPPKGGHHHHHH
**B_1**<br>MDYDIPTTENLYFQGAMVDTLSGLSSEQGQSGDMTIEEDSATHIKFSKRDEDGKELA<br>GATMELRDSSGKTISTWISDGQVKDFYLYPGKYTFVETAAPDGYEVATAITFTVNEQG<br>QVTVNGKATKGDAHIGSAGSAAGSGEFGGKLGSIEFIKVNKGSAGSAAGSGEFGGDY<br>DIPTTENLYFQGAMVDTLSGLSSEQGQSGDMTIEEDSATHIKFSKRDEDGKELAGATM<br>ELRDSSGKTISTWISDGQVKDFYLYPGKYTFVETAAPDGYEVATAITFTVNEQGQVTV<br>NGKATKGDAHIGGHHHHHH
**B_2**<br>MKPLRGAVFSLQKQHPDYPDIYGAIDQNGTYQNVRTGEDGKLTFKNLSDGKYRLFE<br>NSEPAGYKPVQNKPIVAFQIVNGEVRDVTSIVPQDIPATYEFTNGKHYITNEPIPPKGSA<br>GSAAGSGEFGGAHIVMVDAYKPTKGSAGSAAGSGEFGGKPLRGAVFSLQKQHPDYP<br>DIYGAIDQNGTYQNVRTGEDGKLTFKNLSDGKYRLFENSEPAGYKPVQNKPIVAFQIV<br>NGEVRDVTSIVPQDIPATYEFTNGKHYITNEPIPPKGGHHHHHH

Table S2. Amino acid sequences of the constructed proteins used for the functionalization of protein dendrimer. Blue, green, purple and red sequences indicate an EGFR-specific repebody, eGFP, TDP and gelonin, respectively. Green and blue underlined sequences represent SpyTag and SnoopTag, respectively. For the off-target cargos, the sequence of an EGFR-specific repebody is replaced with that of an off-target repebody.

$\mathbf{T_{mono}}$<br>METITVSTPIKQIFPDDAFAETIKANLKKKSVTDAVTQNELNSIDQIIANNSDIKSVQGI<br>QYLPNVRYLALGGNKLHDISALKELTNLTYLMLHYNQLQILPNGVFDKLTNLKELYL<br>SENQLQSLPDGVFDKLTNLTELDLSYNQLQSLPEGVFDKLTQLKDLRLYQNQLKSVP<br>DGVFDRLTSLQYIWLHDNPWDCTCPGIRYLSEWINKHSGVVRNSAGSVAPDSAKCSG<br>SGKPVRSIICPTASGSAGSAAGSGEFGGAHIVMVDAYKPTKGSSKLGSIEFIKVNKGSL<br>ENHHHHHH
$\mathbf{F_{mono}}$<br>MVSKGEELFTGVVPILVELDGDVNGHKFSVSGEGEGDATYGKLTLKFICTTGKLPVP<br>WPTLVTTLTYGVQCFSRYPDHMKQHDFFKSAMPEGYVQERTIFFKDDGNYKTRAEV<br>KFEGDTLVNRIELKGIDFKEDGNILGHKLEYNYNSHNVYIMADKQKNGIKVNFKIRH<br>NIEDGSVQLADHYQQNTPIGDGPVLLPDNHYLSTQSALSKDPNEKRDHMVLLEFVTA<br>AGITLGMDELYKASGSAGSAAGSGEFGGAHIVMVDAYKPTKGSSKLGSIEFIKVNKG<br>SLENHHHHHH
$\mathbf{D_{mono}}$ **(+)**<br>METITVSTPIKQIFPDDAFAETIKANLKKKSVTDAVTQNELNSIDQIIANNSDIKSVQGI<br>QYLPNVRYLALGGNKLHDISALKELTNLTYLMLHYNQLQILPNGVFDKLTNLKELYL<br>SENQLQSLPDGVFDKLTNLTELDLSYNQLQSLPEGVFDKLTQLKDLRLYQNQLKSVP<br>DGVFDRLTSLQYIWLHDNPWDCTCPGIRYLSEWINKHSGVVRNSAGSVAPDSAKCSG<br>SGKPVRSIICPTEFGGGSGGGSGGGSGGASLAALTAHQACHLPLETFTRHRQPRGWE<br>QLEQCGYPVQRLVALYLAARLSWNQVDQVIRNALASPGSGGDLGEAIREQPEQARL<br>ALTLAAAESERFVRQGTGNDEAGAANGSGGGSGGGSGGGSGGTSVSKGEELFTGVV<br>PILVELDGDVNGHKFSVSGEGEGDATYGKLTLKFICTTGKLPVPWPTLVTTLTYGVQC<br>FSRYPDHMKQHDFFKSAMPEGYVQERTIFFKDDGNYKTRAEVKFEGDTLVNRIELKG<br>IDFKEDGNILGHKLEYNYNSHNVYIMADKQKNGIKVNFKIRHNIEDGSVQLADHYQ<br>QNTPIGDGPVLLPDNHYLSTQSALSKDPNEKRDHMVLLEFVTAAGITLGMDELYKKD<br>ELGGSGSGFLGGGSGSAHIVMVDAYKPTKGSSKLGSIEFIKVNKGSLENHHHHHH

G_{mono} (+)

METITVSTPIKQIFPDDAFAETIKANLKKKSVTDAVTQNELNSIDQIIANNSDIKSVQGI
QYLPNVRYLALGGNKLHDISALKELTNLTYLMLHYNQLQILPNGVFDKLTNLKELYL
SENQLQSLPDGVFDKLTNLTELDLSYNQLQSLPEGVFDKLTQLKDLRLYQNQLKSVP
DGVFDRLTSLQYIWLHDNPWDCTCPGIRYLSEWINKHSGVVRNSAGSVAPDSAKCSG
SGKPVRSIICPTEFGGGSGGGSGGGSGGASLAALTAHQACHLPLETFTRHRQPRGWE
QLEQCGYPVQRLVALYLAARLSWNQVDQVIRNALASPGSGGDLGEAIREQPEQARL
ALTLAAAESERFVRQGTGNDEAGAANGSGGGSGGGSGGGSGGTSLDTVSFSTKGAT
YITYVNFLNELRVKLKPEGNSHGIPLLRKKCDDPGKCFVLVALSNDNGQLAEIAIDVT
SVYVVGYQVRNRSYFFKDAPDAAYEGLFKNTIKTRLHFGGSYPSLEGEKAYRETTEL
GIEPLRIGIKKLDENAIDNYKPTEIASSLLVVIQMVSEAARFTFIENQIRNNFQQRIRPAN
NTISLENKWGKLSFQIRTSGANGMFSEAVELERANGKKYYVTAVDQVKPKIALLKFV
DKDPKKDELGGSGSGFLGGGSGSAHIVMVDAYKPTKGSSKLGSIEFIKVNKGSLENH
HHHHH

Off-target repebody (-)

ETITVSTPIKQIFPDDAFAETIKANLKKKSVTDAVTQNELNSIDQIIANNSDIKSVQGIQ
YLPNVRYLALGGNKLHDISALKELTNLTYLILTGNQLQSLPNGVFDKLTNLKELVLVE
NQLQSLPDGVFDKLTNLTYLNLAHNQLQSLPKGVFDKLTNLTELDLSYNQLQSLPEG
VFDKLTQLKDLRLYQNQLKSVPDGVFDRLTSLQYIWLHDNPWDCTCPGIRYLSEWIN
KHSGVVRNSAGSVAPDSAKCSGSGKPVRSIICPT

[1] X. Chen, J. L. Zaro, W. C. Shen, Adv Drug Deliv Rev 2013, 65, 1357.
[2] H. Y. Kim, J. A. Kang, J. H. Ryou, G. H. Lee, D. S. Choi, D. E. Lee, H. S. Kim, Acs Chem Biol 2017, 12, 2891.
[3] J. J. Lee, H. J. Choi, M. Yun, Y. Kang, J. E. Jung, Y. Ryu, T. Y. Kim, Y. J. Cha, H. S. Cho, J. J. Min, C. W. Chung, H. S. Kim, Angew Chem Int Edit 2015, 54, 11020.
[4] S. C. Lee, K. Park, J. Han, J. J. Lee, H. J. Kim, S. Hong, W. Heu, Y. J. Kim, J. S. Ha, S. G. Lee, H. K. Cheong, Y. H. Jeon, D. Kim, H. S. Kim, Proc Natl Acad Sci U S A 2012, 109, 3299.
[5] C. J. Crasto, J. A. Feng, Protein Eng 2000, 13, 309.
